# Found in Translation: Exporting Patient-Centered Communication and Small Group Teaching Skills to China

**DOI:** 10.3885/meo.2009.T0000136

**Published:** 2009-06-26

**Authors:** Benjamin Blatt, Gene Kallenberg, Forrest Lang, Patrick Mahoney, JoEllen Patterson, Beverly Dugan, Shaobang Sun

**Affiliations:** * George Washington University School of Medicine, Washington, DC; † University of California, San Diego, San Diego, CA; ‡ East Tennessee State University, Johnson City, TN; § University of San Diego, San Diego, CA; ǁ Human Resources Research Organization (HumRRO), Alexandria, VA

**Keywords:** Cross-cultural faculty development, doctor-family-patient communication, doctor-patient relationship, China, small group teaching

## Abstract

The Chinese Medical Doctor's Association asked us to develop a train-the-trainers program in doctor-patient communication and in teaching skills for a select group of Chinese health care professionals, who would then serve as trainers for practicing physicians throughout China. The request came in the context of increasing doctor-patient friction related, in part, to the dissolution of the socialist health care safety net in China. In this article we recount the implementation of our 5-day training program in Beijing. We explore cross-cultural issues that arose in presenting the program's two principal training domains: small group teaching and patient-centered doctor-patient communication. We also explore the linguistic challenges we encountered as non-Chinese speaking teachers. Finally, we reflect on the lessons learned from this project that may be of value to others called upon to export Western doctor-patient communications training to other cultures. In this age of increasing globalization, cross-cultural sharing of medical education represents a growing trend.

## I. The Challenge

*“On the afternoon of April 17 [2001], Wang Kai, physician in charge of the No. 1 hospital affiliated with the Huaxi Medical University, was stabbed 13 times by He Haijun, one of his patients”*.^1^

*“Only 6.8 per cent of doctors said the relationship between patient and doctor is harmonious; and 26.8 per cent could not understand why the patient and their families were so uncooperative”*.^2^

Our group was asked to plan a train-the-Trainers course in Western-style doctor-patient communication skills for a select group of Chinese health professionals (the Trainers) who would in turn train practicing physicians throughout China. This request was initiated by an American human resources development firm (Human Resources Research Organization—HumRRO) working in partnership with the Chinese Medical Doctors Association (CMDA), a quasi-governmental AMA-like organization of 2 million Chinese physicians.

Worsening communications in the Chinese medical system date from 1979 when the socialist health care safety-net was replaced by an underfunded fee-for-service system.^3^ A survey conducted by the Beijing Doctors’ Association in 2001 identified 502 attacks on medical staff, resulting in ninety disabling or serious injuries.^1^ Such sensational examples have contributed to the realization that the traditional hierarchical approach to the doctor-patient relationship used by current Chinese physicians is increasingly inadequate.^4^

CMDA and the Chinese government decided to take action to address China's medical communications problems. Though improved communication skills training had already entered Chinese medical school curricula,^5^ our effort, coupled with a national distribution system sponsored by the CMDA, would have the potential to rapidly reach many Chinese physicians already in practice. Involving Americans would provide the caché and authority of Western expertise which currently carries great weight with Chinese leaders in their efforts to create a more modern society.

Through a Chinese professional training company as intermediary, CMDA connected with Dr. Shaobang Sun, a Chinese national working with HumRRO, to bring our group to China. They devised a business plan where HumRRO would advance funding for the cost of program development. Once trained, the select group of Chinese doctors would offer the program for a fee as continuing medical education to other doctors throughout China and eventually reimburse HumRRO with a portion of the profits.

## II. Program Objectives and Development

During 2005–2006, we crafted a five-day train-the-Trainer program in doctor-patient communication for CMDA, adding at our discretion a major section on Western small group teaching-skills. Finally, per CMDA specification, we included a section on ethics. Our objectives were to provide the Trainers with an adequate level of knowledge and skills to train other physicians in a Western approach to doctor-patient communication and ethical analysis. To support the course we created manuals and a series of illustrative videos, either in Mandarin or in English with Chinese subtitles. At CMDA's request, Dr. Sun created a complementary short text. Essential to their business model, the text combined with the course would provide sufficient hours for Chinese doctors to fulfill state CME requirements. Concerned that an externally developed program might not be met with approval in China, we advocated for conducting a needs assessment and for sharing decision making through direct contact (e.g., web camera conference calls). CMDA preferred that we submit a proposal and that Dr. Sun serve as the feedback conduit.

The Western faculty (“the faculty”) developed the program over the course of a year in consultation with Dr. Steven Cole, author of a well known interviewing text.^6^ Drafts were exchanged and a final version agreed upon. Three months before going to China, the faculty met in Washington, DC to rehearse the program.

**Figure 1.**
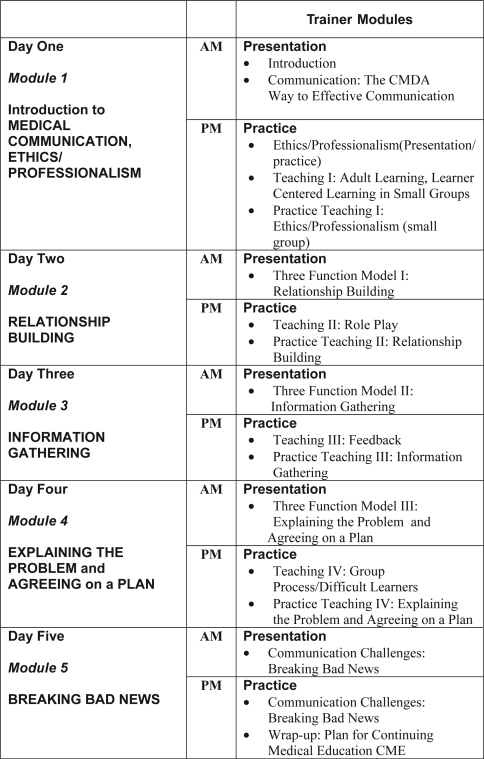
Summary of the Train-the-Trainers Program

Our strategy for training the Trainers was straightforward. In the mornings they would experience the course in the identical way their future learners would, through presentations and role plays (“see one, do one”). In the afternoons, after teaching-skills reviews, they would practice-teach the morning communication topics (“teach one”) (Figure 1). Throughout the program, we emphasized the similarities of American and Chinese social evolution: both fueled by the rise in consumerism, patient access to on-line medical information and a movement from doctor-centered to a more patient-centered relationship.^3^,^7^,^8^,^9^,^10^

Though explicitly invited to bring a Western communication approach to China, we initially tried to incorporate Chinese culture-bridging components into the program, titling it the “Harmony Course in Doctor-Patient-Family Communication” and using the Daoist yin-yang symbol as the program logo. Translating the manual into Chinese, CMDA eliminated these culture-bridging attempts. Further, during a preliminary publicity trip to China they would not accept one of our faculty, a Chinese-American doctor, as anything but a translator. CMDA seemed to want the program to have a Western look.

## III. Program Implementation in China

In planning the program we anticipated challenges from differences in language and in East-West approaches to our program's two principal training areas: small group teaching and doctor-patient communication.

Regarding teaching, we expected resistance to our planned use of non-lecture-based education formats, the lecture being the standard Chinese teaching modality. Culture-based reticence, formality, politeness norms, concern with losing face, and tradition-bound respect for us as teachers might inhibit the Trainers from participating actively in role plays and from expressing their personal feelings about charged issues.

We were also concerned about resistance to our patient-centered communication methods, which emphasized individual autonomy. The traditional Chinese approach to the doctor-family-patient relationship reflects an authority hierarchy with the doctor on top, then family, then individual. The concept of “person” in China is not rights-oriented but duty-oriented in order to support this hierarchy.^4^

### Cross Cultural Challenges: Language Barriers

Only 24 hours before the program's start, we met our Chinese translators for the first time and spent six hours rehearsing with them. On Day One of the program, after a glitzy opening ceremony with banners, speeches and picture-taking, we addressed 49 medical professionals, most of whom had no or rudimentary spoken English skills.

The greatest challenge was in the small group skill-building workshops for which role-plays were central. Realizing that word-for-word translation would be cumbersome enough to impair the flow of learning,^11^,^12^ we opted for periodic summaries. This edited “big picture translation” required practice and deft interpretation to provide the right mix of meaning and detail. Fortunately, our translators were highly skilled, most having trained in a Beijing university baccalaureate degree program in English-Chinese medical translation. To avoid distracting the group, the translation was whispered into the ear of the western faculty leader. From a distance it looked like the faculty leader and translator were “joined at the head.” While intervals between summary translations varied widely, on average these occurred every 1–2 minutes. At first awkward for us, this method soon became second nature.

### Cross Cultural Challenges: Small Group Education

#### Interactive Small Group Learning

At 5:00 PM of Day One in a plenary feedback session, Trainers, exposed to small group learning for the first time that afternoon, clamored for less small group learning and more “theory and references.” On Day Two during small group role play, Trainers in some of the groups provided their colleagues with lengthy criticisms rather than giving balanced feedback per our instruction.

Day Three, however, was different. Dramatic change occurred, often initiated by the modeling of older Trainers with prestigious backgrounds. When teaching, Trainers now praised the strengths of their learners before moving to corrective suggestions. Simultaneously, the relationship between Trainers and Western faculty, at first respectful but guarded, became warm and collegial as we explored the role-play situations together. Upon returning to our skills groups, we found stacks of course manuals awaiting us with requests from Trainers to sign their books next to our names and to have photos taken with them.

#### Participation

Anticipating that culture-based politeness norms would result in token participation, we were delighted by the robust response. Our challenge was not in eliciting it but in containing it. On one typical morning in plenary session, we measured Trainers’ comment time: no speaker finished in less than 2.5 minutes. Many of the responses actually were expanded restatements of points made by our communication faculty during the presentation: “authoritative” confirmation of the points of the day, as if the speakers were vying for the respect of peers.

### Cross Cultural Challenges: Doctor-Family-Patient Communication

#### Role of the Doctor

##### Traditional Authority

Initially, Chinese physicians responded in a hierarchical manner to role-play patients questioning their judgments: “I'm your doctor, and I'm telling you to do this. Don't question me.” Further, despite our presentation of patient-centered principles, Chinese doctors initially confined themselves to exhaustive closed-ended diagnostic questioning of these “patients.”

By the third day, however, simultaneous with their use of learner-centered teaching techniques, Trainers implemented empathy and active listening in their role plays and explored patients’ concerns regarding their illness. If they relapsed into authoritarian mode, they laughed at themselves and self-corrected. One Chinese gastroenterologist who changed his style proudly told us about how patient-centeredness had always been part of his practice, noting that he himself had carried soup made by his wife to a poor patient.

##### Strong Emotions

Cultural differences surfaced in the comfort of Chinese and Western doctors in communicating with patients about end of life situations,^13^ depression,^14^ suicide, and deeply personal emotions.

In one role play, when a “depressed patient” shared traumatic stories about his family life and loss, the Chinese Trainers were shocked: “This would never happen in China. The patient is reporting details that are private and should only be shared within the family, if at all, not with the doctor.” Many Trainers communicated that physicians were not trained to talk about depression and suicide, though aware they are significant problems in China.^14^,^15^,^16^ Other participants suggested that strong, painful emotions are difficult for both the doctor and the patient, would only lead to awkward silences, and were better left unarticulated. Not all Trainers, however, were uncomfortable discussing emotionally charged patient issues. In several groups, members even discussed increasingly personal stories about themselves: the death of a husband (blamed in part on the medical system) and the difficulties of caring for a father with lung cancer. For Trainers uncomfortable with personal conversations, however, our program did not appear to increase their comfort level.

#### Role of the Family

##### Medical Decision Maker

Trainers reminded us on several occasions that traditional Chinese beliefs emphasize family role over patient autonomy^4^ in deciding whether to disclose information to the patient: “You know, here in China,” stated a Trainer, “we believe that it is appropriate to tell the patient's family about the condition and allow them to decide how best to handle the patient's informational and emotional needs.” Fortunately, we designed our practice cases as doctor-family-patient dialogues, so this East-West difference did not constitute a training barrier.

In one of the skills groups, the faculty leader asked the Trainers about their own personal preferences. Surprisingly, all nine participants expressed a preference to have medical information communicated to them directly, as did 48/49 Trainers when the whole group was polled later. “Of course I wouldn't receive that information first,” stated one Trainer, “because in China no one would approach me first.”

##### Advocate and Protector

Since some Chinese patients distrust their doctors, assuming their doctors are willing to exploit them for financial gain, they turn to their family for protection. While the family is expected to protect the patient's interests, the Trainers shared stories of the patient protecting the family's needs: patients choosing suicide to protect their family from costly medical expenses.

## IV. Evaluation of the Course by Chinese Doctors

We informally carried out content analysis of the Trainers’ narrative reports describing their reactions to the workshop. We first identified categories of responses and then grouped the categories into overarching categories, or meta-categories (Table 1). Thirty-two comments indicated that the interactive teaching process was the program's most impressive feature. Perhaps out of politeness, there were no negative comments on the form. Constructive feedback, however, was presented to us orally: our role-play cases needed to be more “Chinese,” and government policies would have to change substantially in order to support the principles learned in the course.

**Table 1.** Summary of Participants’ Narrative Reports (N = 49)

Meta-Category	Category	Number of Comments	Example
Gain from the workshop	*Attitude change/awareness improvement*	35	“I have a better understanding of the importance of doctor-patient communication.”
	*Knowledge and skills*	22	“I have learned basic knowledge and skills of doctor-patient communication.”
	*Teaching skills*	14	“My teaching skills have been improved.”
Most impressive things	*Interactive teaching process*	32	“The teaching process made our learning very interactive, interesting and enjoyable.”
	*Professional teachers*	19	“Our American teachers are excellent role models.”
Other comments	*Commitment to the future*	20	“I would like to make my contribution in this area.”
	*Overall reactions*	16	“The workshop was very successful.”
	*Cultural adaptation*	10	“We need to consider some unique characteristics of our culture when teaching in the future.”
	*Being proud*	6	“I feel honored.”
	*Government policy*	4	“Government's support at the policy level is important to ensure the success of this training program.”

## V. Lessons Learned

In the presentation of our train-the-Trainers program in communications and teaching skills to 49 Chinese health care professionals, East-West cultural differences presented challenges in three major domains: language, teaching methods and doctor-patient communication. The lessons learned include the following six points.

1. Whispered summary translation is an effective method of small group teaching in the presence of language barriers.

This “brain-sharing” between faculty and translators resulted in effective role play and small group experiential education with Chinese learners. Keys to this success were interpreters with university-based medical translating skills and their clear understanding of the course's objectives, achieved by an intense rehearsal of the entire program.

2. Personal experience-sharing opens the door to small group learning with learners from a lecture-based education tradition.

Faculty modeling (we shared our own personal experiences) plus role play resulted in Trainers reciprocally and animatedly sharing their own experiences as care-givers and patients. Including everyone as role-player and then observer/critic in a safe learning climate resulted in Trainers examining their own emotions and those of their patients. This learning climate, which safely fostered personal involvement, seemed to ignite Trainers’ enthusiasm for small group learning. Buy-in from older, high-status group members, which occurred early in the process, may have also aided in acceptance. In addition, the aura of “things American” and the Hawthorne effect (behavior change following increased attention) may have also contributed. Vigorous participation, however, had some drawbacks, especially with professionals used to communicating in long speeches. Clear boundaries needed to be established. Once in place, they were willingly respected.

3. Cultural traditions, even those that seem to contradict our own, should be explored for common ground.

There is a shared set of truths and conventions between all societies and their relationships with their “healers” that can serve as cross-cultural bridges. As in all cultures, Chinese medical tradition contains multiple, sometimes contradictory, themes that can be activated or suppressed by contextual pressures. Though doctor-patient tension in China was fostered by a hierarchical communication tradition in the context of an under-responsive new health care system, other more humanistic strains exist in Chinese medical tradition. They are reflected in the work of Sun Simao (AD581-682), for example. The author of the first full-length explicit statement on medical morality, he wrote, “Treat patients as if they were your own family member.” They are also present in Confucian teaching, which emphasizes that the purpose of medicine is to “treat people by love”^4^. Our training method may have provided implicit permission for the Trainers to tap into these more humanistic elements.

As it is worthwhile to search the past for themes-in-common when developing a cross-cultural educational program, it is also worthwhile to look to the future. Our thematic emphasis on similarities in evolution of Chinese and American society may have contributed to the program's acceptance. We emphasized forces pushing us in similar directions that come with advancing economic and living standards of modern societies.^17^ These include patients’ internet access to scientific information, the demands of an emerging consumer middle class, and the transition of leading medical problems from acute infectious and traumatic illnesses to chronic illnesses. These forces are already opening China to a culturally different (and for us familiar) medical communications style.

4. Opening a dialogue about deep feelings with learners from a reticent culture is worthwhile.

Our experience with Chinese doctors’ willingness to explore their patients’ and their own deeper feelings was embraced by some and rejected by others. Because of some very positive responses, however (which included sharing of highly personal stories), we would recommend opening a dialogue about this subject in any cross-cultural doctor-patient communications program. It offers opportunities to develop relationship-building skills in willing participants by revealing the direct parallels between their own feelings and those of their patients.

5. Cross-cultural teachers should seek opportunities for bi-directional learning.

What we learned from our colleagues about the special role of family in Chinese health care confirmed our long-held impressions that family support and advocacy are undervalued in Western medicine and even inhibited by our cultural emphasis on individualism. We came away both admiring this strength of Chinese society and reflecting about how to better incorporate the Chinese inclusion of family in our own interaction with patients

6. Cross-cultural exchanges require political astuteness and guidance from a bi-cultural intermediary.

Cross-cultural interactions can be complicated. As previously noted, our attempts to integrate certain Chinese features into the course were resisted, not welcomed, since CMDA wanted to develop and sell an American product. We achieved more understanding of this issue than several other issues involving CMDA. We never understood CMDA's selection process for Trainers and their incentives for participation (some had strong connections with pharmaceutical companies). We also never understood CMDA's reluctance to collaborate on a pre-program needs assessment and a post-program follow-up plan. Misinterpretations and differences in approach might have undermined our collaboration had Dr. Sun not served as our advisor and ambassador. As others have found,^18^ a bilingual, bicultural, well-connected intermediary is crucial to the success of a cross-cultural program.

### Post Script

As of this date, the communications course has been implemented by CMDA in a number of locations in Guangdong, Hebei, Shandong, and Sichuan provinces. About 500 doctors/physicians have attended the training courses. To meet the need for more instructors, CMDA recently conducted two train-the-Trainer workshops by using our program's graduates as trainers.

Because the training course has received highly positive comments from trainees, CMDA now values it one of its brand products. Nonetheless, CMDA officials indicate that dissemination of the program has met with some resistance: for many hospitals and doctors, doctor-patient communication skills are still considered “soft” as compared to medical skills, and they are somewhat reluctant to pay for learning them.

### Conflicts of Interest

The authors declare no conflicts of interest.
